# Presenilin-1 Dependent Neurogenesis Regulates Hippocampal Learning and Memory

**DOI:** 10.1371/journal.pone.0131266

**Published:** 2015-06-22

**Authors:** Jacqueline A. Bonds, Yafit Kuttner-Hirshler, Nancy Bartolotti, Matthew K. Tobin, Michael Pizzi, Robert Marr, Orly Lazarov

**Affiliations:** 1 Graduate Program in Neuroscience, University of Illinois at Chicago, Chicago, Illinois, 60612, United States of America; 2 Department of Anatomy and Cell Biology, College of Medicine, University of Illinois at Chicago, Chicago, Illinois, 60612, United States of America; 3 Medical Scientist Training Program, University of Illinois at Chicago, Chicago, Illinois, 60612, United States of America; 4 Midwestern University, 555 31^st^ street, Downers Grove, IL, 60515, United States of America; 5 Department of Neuroscience, Rosalind Franklin University of Medicine and Science, North Chicago, Illinois, 60064, United States of America; CSIC/Universidad Autonoma Madrid, SPAIN

## Abstract

Presenilin-1 (PS1), the catalytic core of the aspartyl protease γ-secretase, regulates adult neurogenesis. However, it is not clear whether the role of neurogenesis in hippocampal learning and memory is PS1-dependent, or whether PS1 loss of function in adult hippocampal neurogenesis can cause learning and memory deficits. Here we show that downregulation of PS1 in hippocampal neural progenitor cells causes progressive deficits in pattern separation and novelty exploration. New granule neurons expressing reduced PS1 levels exhibit decreased dendritic branching and dendritic spines. Further, they exhibit reduced survival. Lastly, we show that PS1 effect on neurogenesis is mediated via β-catenin phosphorylation and notch signaling. Together, these observations suggest that impairments in adult neurogenesis induce learning and memory deficits and may play a role in the cognitive deficits observed in Alzheimer’s disease.

## Introduction

New neurons incorporate in the granular layer of the dentate gyrus (DG) of the hippocampus throughout life [1]. It is estimated that in the human brain, 700 neurons are added in each hippocampus per day. Further, about one third of the neurons in the DG are subject to exchange [2]. New neurons in the adult hippocampus are thought to play a role in forms of learning and memory. However, the extent to which neurogenesis contributes to this process is unknown [3, 4]. Further, whether deficits in neurogenesis play a role in cognitive dysfunction is not clear. This is partially due to the difficulty to discriminate between the role of younger and older neurons. Unraveling the role of neurogenesis in hippocampal function will contribute greatly to the understanding of memory formation. In turn, it will provide insight into mechanisms of diseases affecting memory, such as Alzheimer’s disease (AD), the most prevalent form of dementia in the elderly [5].

Mutations in *presenilin-1* (*PSEN1*) cause rare aggressive familial Alzheimer’s disease (FAD). PS1 is the catalytic core of the aspartyl protease γ-secretase [6–12]. We previously reported that PS1 regulates the differentiation of adult NPCs [13]. Results from this study show that injection of lentivirus expressing PS1 shRNA into the subgranular layer (SGL) of the DG decreased proliferation of NPCs and enhanced their differentiation into neurons and glia at 6 weeks following injection. Here, we tested the hypothesis that knockdown of PS1 in hippocampal NPCs would be sufficient for the induction of cognitive deficits. For this purpose, lentivirus expressing either PS1 shRNA or control shRNA was injected bilaterally into the DG of 6 month old C57BL/6 mice as previously described [13]. Our results show that reduced PS1 expression in NPCs and new neurons causes impairments in pattern separation and novel object recognition (NOR). Furthermore, these impairments may be due to defective differentiation of new neurons that results in compromised dendritic morphology and impaired survival.

To gain an insight into the mechanism by which PS1 regulates hippocampal neurogenesis we infected adult neurospheres with our lentivirus expressing either control shRNA or PS1-targeted shRNA and found that downregulation of PS1 expression in NPCs results in reduced levels of phosphorylated β-catenin and notch intracellular cleavage fragments (NICD). Excitingly, addition of exogenous NICD to neurosphere cultures with shRNA-mediated knockdown of PS1 rescues the premature neuronal differentiation phenotype. These observations strongly suggest that impairments in neurogenesis via downregulation of PS1 induce learning and memory deficits and may play a role in the development of Alzheimer’s disease.

## Results

### Knockdown of PS1 in Neural Progenitor Cells in the Subgranular Layer of the Dentate Gyrus Induces Learning Impairments in Pattern Separation and Exploratory Preference

**Fig 1:**
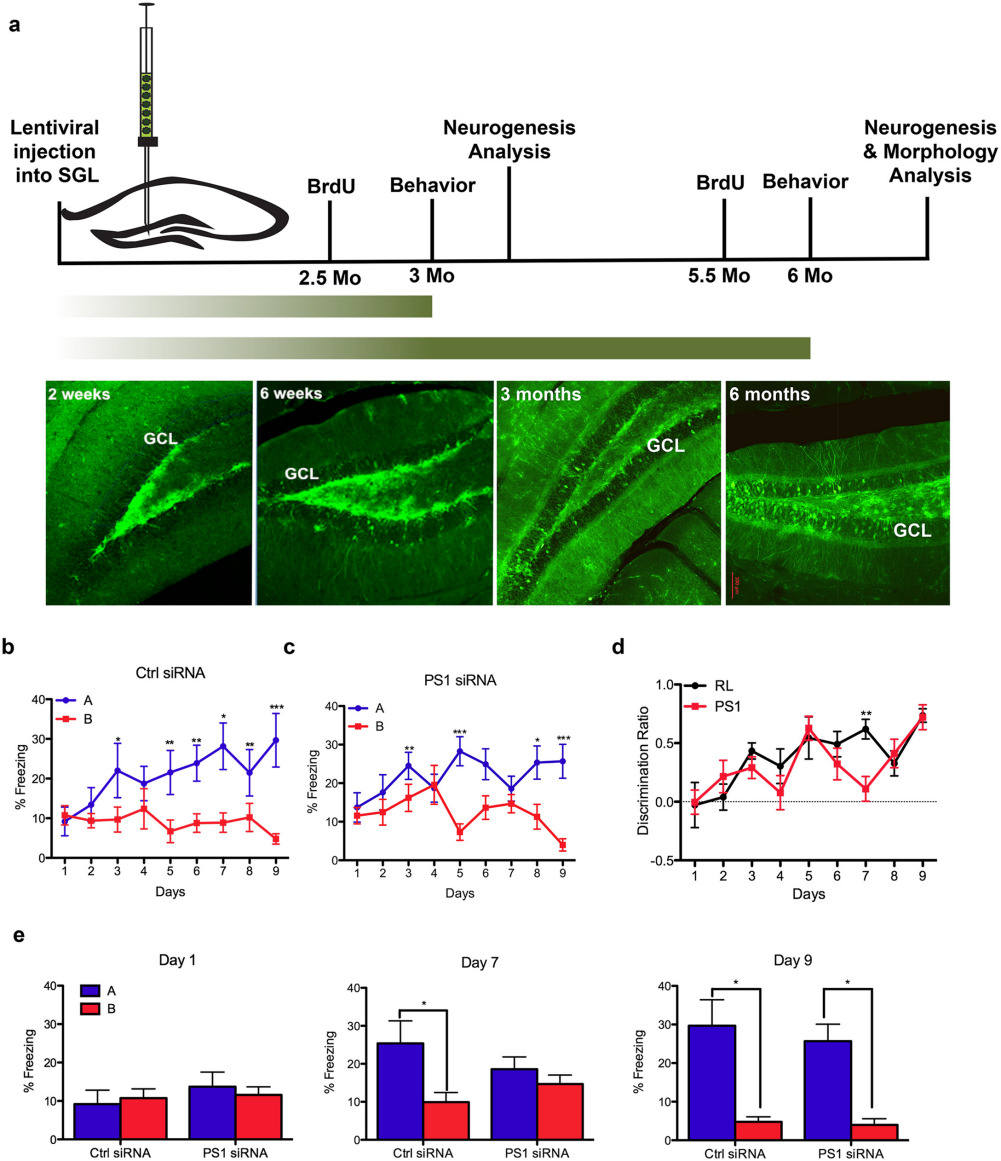
Impairments in pattern separation 3 months after PS1 knockdown in neural progenitor cells. **A**. Experimental timeline. GFP expression in the dentate gyrus of the hippocampus at 2 weeks, 6 weeks, 3 and 6 months after lentivirus injection. Two weeks following injection, GFP-infected cells are located in the subgranular layer of the dentate gyrus. Migration of GFP+ cells into the granular cell layer is apparent at 6 weeks post injection, while at 3 and 6 months post-injection many GFP^+^ cells are incorporated in the granular cell layer. **B-C** Percentage of freezing in context A (shock) vs. context B (similar context) of mice 3 months following injection with either control (15) shRNA (**B**) or with PS1 shRNA (**C**) (two-way repeated measures (context and days) ANOVA, F_(context)1,16_ = 5.02, P<0.05; and F_(context)1,18_ = 6.18, P<0.05 respectively). Paired t-test, *p<0.05, **p<0.005. Error bars indicate ± SEM. **D**. Discrimination ratio [(A-B)/max(A,B)] between the control and the PS1 injected animals (two-way ANOVA [treatment and days] with repeated measures, F_(treatment)1,153_ = 1.38, P>0.05) Error bars indicate ±SEM. **E**. Post-hoc comparisons between contexts and treatments on single days during the recognition memory paradigm (paired t-test, *p<0.05).

To examine whether the function of new neurons in hippocampal learning and memory is PS1-dependent, lentivirus vectors expressing either control shRNA or PS1 shRNA were injected into the SGL of the DG. We observed infected NPCs expressing green fluorescent protein (GFP^+^) in the SGL of the DG solely at 2 weeks post injection while the granular cell layer (GCL) was free of GFP^+^ cells (Fig 1a). By 6 weeks, GFP^+^ NPCs were apparent in the GCL, but the vast majority was still detected in the SGL (Fig 1a). At 3 and 6 months after injection, most of the GFP^+^ cells were detected in the GCL while the relative amount of GFP^+^ cells in the SGL was reduced (Fig 1a). Given the time-dependent increased contribution of infected new neurons to the GCL, we examined the learning and memory capabilities of these mice 3 and 6 months post injection, followed by analysis of NPC populations and new neurons expressing reduced levels of PS1.

First, we examined the performance of these mice in pattern separation, a task known to be neurogenesis-dependent [14]. Both control shRNA and PS1 shRNA injected mice exhibit a significant interaction between both contexts and days (Fig 1b and 1c; F_1,16_ = 5.02, *P<0.05, N = 9 and F_1,18_ = 6.203, *P<0.05, N = 10, respectively) 3 months after injection. To test the discrimination on each day, paired t-test was performed comparing the percentage of freezing in the two contexts. Significant differences were found on days 3 (*P<0.05), 5, 6, 7, 8 and 9 (all **P<0.005) in the RL shRNA injected mice (Fig 1b and 1e). Significant context discrimination differences were found on days 3, 5 (**P<0.005), 8 (*P<0.05) and 9 (**P<0.005) in the PS1 shRNA injected mice (Fig 1c and 1e). Analysis of discrimination ratios for each group over the 9 days of testing reveals no significant difference at this time point (Fig 1d, F_(treatment)1,17_ = 0.57, P>0.05).

**Fig 2:**
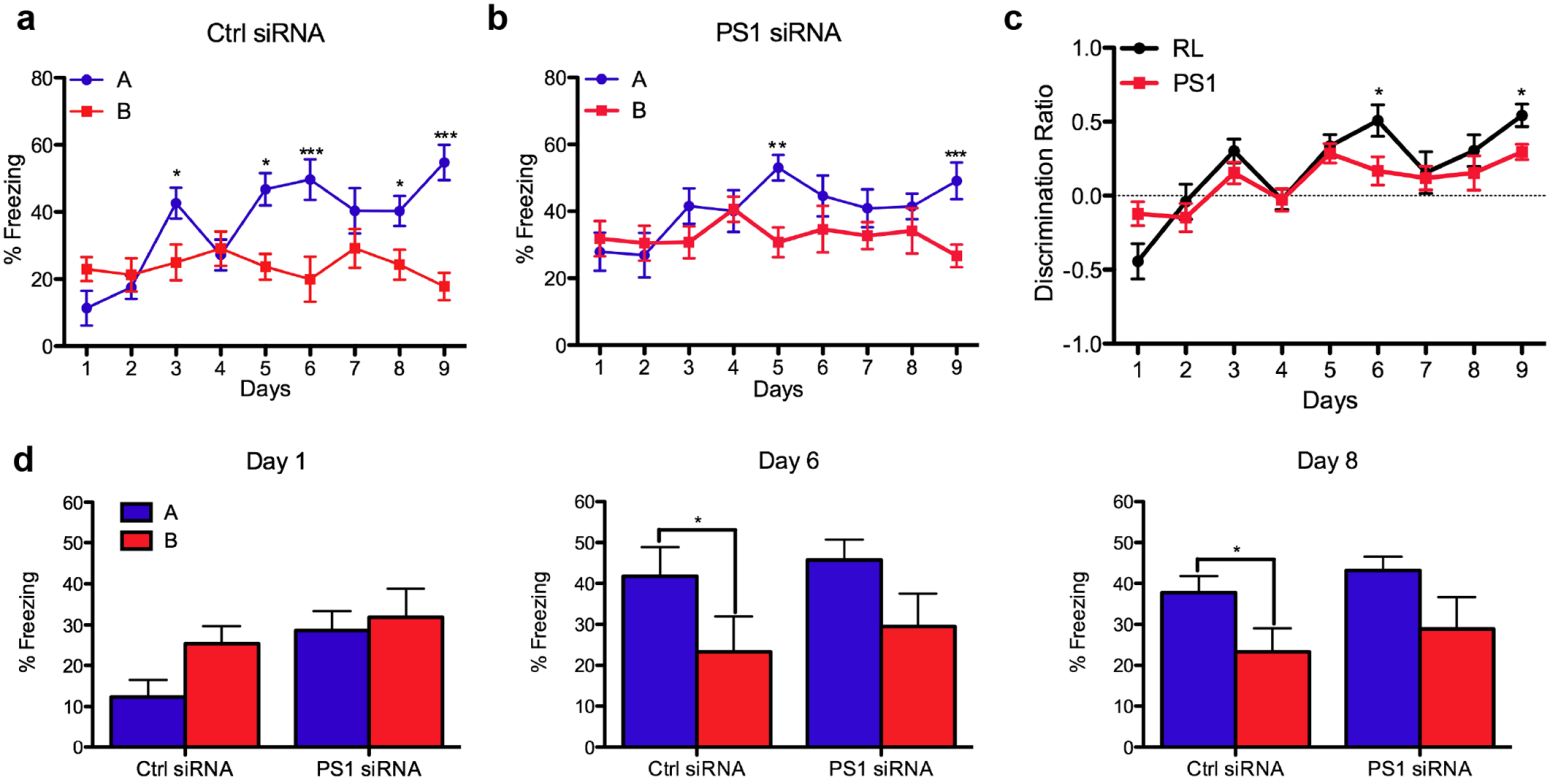
Progressive impairments in pattern separation 6 months after PS1 knockdown in neural progenitor cells. Percentage of freezing in context A (shock) vs. context B (similar context) of 14 month old mice, 6 months following injection with either control shRNA (**A**) or with PS1 shRNA (**B**) (two-way repeated measures (context and days) ANOVA, F_(context)1,14_ = 6.92, P<0.05 and F_(context)1,14_ = 1.88, P>0.05, respectively). Paired t-test, *P<0.05, **P<0.005. Error bars indicate ± SEM. **C**. Discrimination ratio [(A-B)/max(A,B)] analysis reveals a significant difference between the RL shRNA and PS1 shRNA injected animals (two-way ANOVA (treatment and days) with repeated measures, F_(treatment)1,126_ = 3.95, *P<0.05). Error bars indicate ±SEM. **D**. Post-hoc comparisons between contexts and treatments on individual days in the recognition memory paradigm (paired t-test, *p<0.05).

To examine whether differences between the groups become more apparent as more infected neurons incorporate in the GCL, we examined the performance of mice in the pattern separation paradigm 6 months after injection. At this time, both RL shRNA mice showed a significant context and days interaction (Fig 2a; F_1,14_ = 6.92, *P<0.05, N = 8) while the PS1 shRNA mice showed no significant difference (Fig 2b; F_1,18_ = 1.88, P>0.05, N = 8). Paired t-test revealed significant differences in context discrimination on days 3, 5, 6, (**P<0.005), 8 (*P<0.05) and 9 (***P<0.0001) in the RL shRNA injected mice (Fig 2a and 2d), while in the PS1 shRNA injected mice differences were found only on days 5 and 9 (**P<0.005, Fig 2b and 2d, respectively). Analysis of discrimination ratio indicates that PS1 shRNA injected mice show delayed and deficient context discrimination compared to the RL shRNA injected mice 6 months following injection (Fig 2c; F_(treatment)1,126_ = 3.95, *P<0.05, N = 8). Furthermore, taking into account the age-related effect in both the RL and PS1 condition (mice were older in the latter group– 12 months compared to 9 months), the PS1 shRNA mice showed greater deterioration.

**Fig 3:**
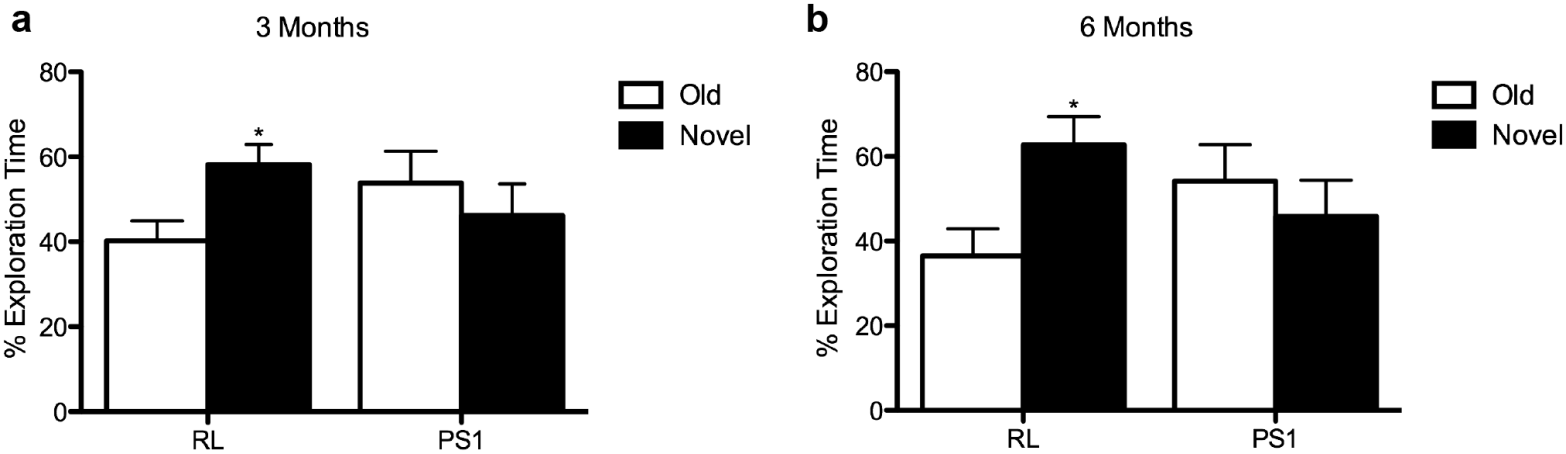
Impairments in novel object recognition at 3 and 6 months after PS1 knockdown in neural progenitor cells. Percentage of time spent exploring the novel or familiar object by mice injected with either the control RL shRNA or PS1 shRNA at 3 **(A)** and 6 months **(B)**. In contrast to RL-injected mice, PS1 shRNA-injected mice show no preference for the novel object at both time-points (unpaired t-test, *p<0.05). Error bars indicate ±SEM.

A common test for the diagnosis of hippocampal amnesia is visual paired comparison task. Novel object recognition (NOR) is the equivalent in rodents. Impairments in NOR have been shown in FAD-linked mouse models [14]. Thus, we attempted to determine whether PS1 knockdown in new neurons has an effect on exploratory preference. We observed that RL shRNA injected mice showed a clear preference for the novel object both 3 and 6 months post injection (Fig 3a and 3b; N = 7, *P<0.05), while the exploratory preference of PS1 shRNA mice was impaired, as manifested by their exploration time (Fig 3a and 3b, N = 8, P>0.05). These results suggest that downregulation of PS1 in NPCs and new neurons, has long-term effects on hippocampal function.

### Reduction in PS1 Expression Induces a Decrease in Survival Rate of Newly Incorporated Neurons in the Granular Cell Layer

**Fig 4:**
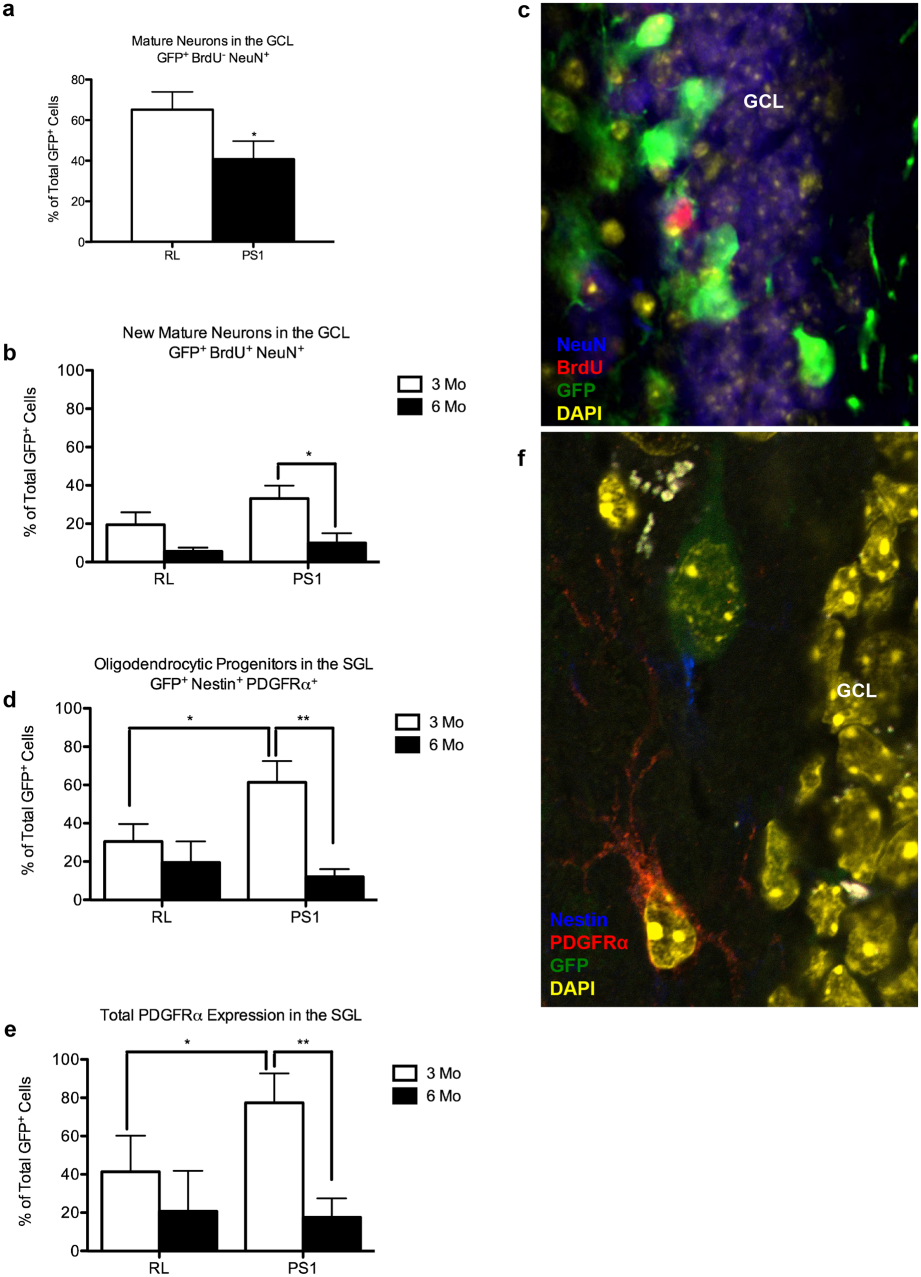
Compromised survival of new neurons expressing reduced levels of PS1 in the granular cell layer of the dentate gyrus of adult mice. Unbiased stereological analysis of green fluorescent protein positive (GFP^+^) cell populations in the SGL and GCL of the DG at 3 and 6 months post-injection. **A**. Less mature neurons within the GCL (GFP^+^BrdU^-^NeuN^+^; *P<0.05) of mice injected with PS1 shRNA. **B**. Separate Comparisons within RL and PS1 groups at 3 and 6 months post injection reveals reduced survival of new neurons in the GCL of PS1 shRNA (GFP^+^BrdU^+^NeuN^+^; *P<0.05). Error bars indicate ±SEM. **C**. Confocal image (Zeiss LSM 510) representing colocalization of GFP with NeuN and BrdU (63x). **(D,E)**. Comparisons within RL and PS1 groups at 3 and 6 months post injection shows reduced rate of survival of PDGFRα^+^ NPCs in the SGL (GFP^+^PDGFRα^+^Nestin^-^; **P<0.01) **(D)** and of total neurons and oligodendrocytes within the SGL (GFP^+^PDGFRα^+^; **P<0.01), **(E)**. Error bars indicate ±SEM. **F**. Confocal image (Zeiss LSM 510) representing colocalization of GFP, nestin and PDGFRα (63x).

To elucidate the mechanism by which reduction in PS1 expression in NPCs and new neurons compromises hippocampal function, we examined the effect of PS1 knockdown on the neurogenic lineages in the DG. We previously showed that 6 weeks following lentiviral injection, there are more new neurons in the SGL of mice injected with PS1 shRNA compared to control shRNA [13]. Thus, we first examined whether this increase is sustained 3 and 6 months post injection. We observed no difference in the number of neuroblasts or immature neurons in the SGL of the two groups 3 and 6 months post infection (S1 Fig 1ai-iii). This may suggest that either their rate of survival is compromised or that the increase in differentiating NPCs is transient. To address the former, we examined the number of new neurons in the GCL. Indeed, we observed that the number of mature neurons in the GCL of mice injected with PS1 shRNA is significantly reduced 3 months post injection (Fig 4a). To verify that their rate of survival is compromised, we compared the number of new neurons in the GCL at 3 months with their number at 6 months. We found that in the control injected mice, there was a reduction in the number of surviving new neurons in the GCL, albeit not statistically significant (Fig 4b and 4c). In contrast, there was a significant reduction in the number of surviving new neurons in the PS1 shRNA group (Fig 4b, *P<0.05). To examine whether NPCs exhibit reduced rate of survival when PS1 is downregulated, we went back and examined the number of surviving NPCs in the SGL at 3 and 6 months post injection in each one of the groups. Indeed, significantly less NPCs (PDGFRα expressing progenitors that give rise to oligodendrocytes or neurons) survived in the SGL of the PS1 shRNA compared to control mice (Fig 4d and 4f, *P<0.05). Likewise, a significant drop in the survival of mature neurons and oligodendrocytes expressing PDGFRα was observed between the 3 and 6 month time points in the PS1 shRNA mice (Fig 4e, *P<0.05). Further analysis revealed no effect on the number of neural stem cells or mature astrocytes (S1 Fig 1bi-iii). Taken together, these results suggest that downregulation of PS1 in NPCs compromises their survival, as well as the survival of new neurons, leading to reduced number of new neurons in the GCL.

### PS1 Knockdown Causes Compromised Morphology of Newly Incorporated Neurons in the Granular Cell Layer

**Fig 5:**
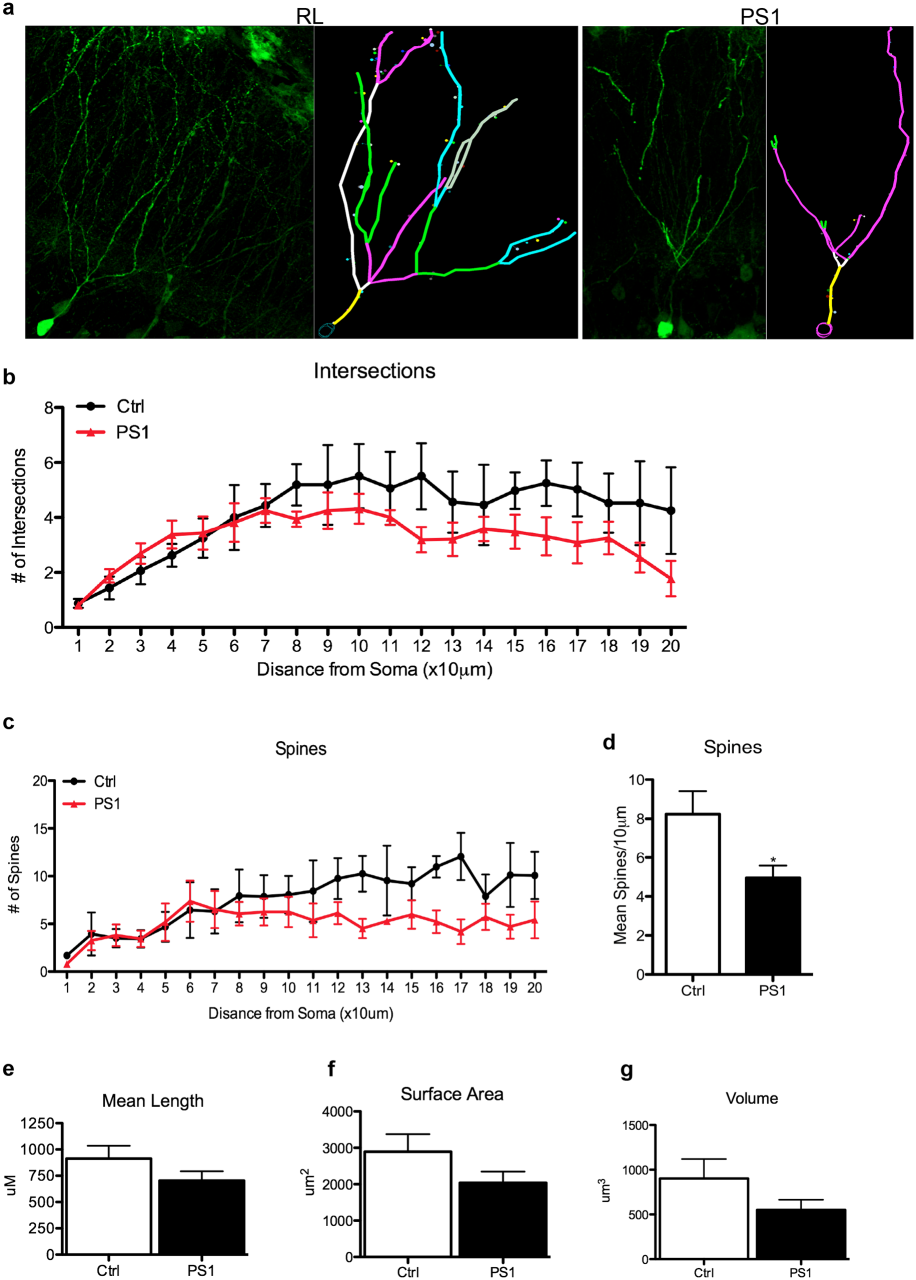
PS1 downregulation in new neurons decreases dendritic arborization and spine density. **A**. Compressed Z-stack confocal images (Zeiss LSM 510) of newly mature GFP^+^ neurons (GFP^+^BrdU+NeuN^+^) in the granular cell layer of the dentate gyrus of RL shRNA- and PS1 shRNA- injected mice 6 months after injection. Images show a dramatic decrease in the number of dendritic arborization in mature GFP^+^ neurons in the PS1 shRNA- injected mice. **B**. Sholl analysis of the number of intersections measured from n = 16 neurons per group. PS1 animals show decreased number of intersections (two-way ANOVA (treatment and distance from soma) F_(treatment)1,120_ = 14.75, P<0.001, F_(distance)19,120_ = 4.10, P<0.0001). **C, D**. PS1 downregulation results in decreased number of spines **(C)** as a function of increasing distance from the soma (two-way ANOVA (treatment and distance from soma) F_(treatment)1,60_ = 13.10, P<0.001, F_(distance)19,60_ = 3.18, P<0.001) and mean spine density **(D)** (unpaired t-test, *P<0.05). **F, G**. Dendritic surface area (**F)** and volume **(G)** were unaffected by PS1 downregulation. Error bars indicate ±SEM.

Next, we examined the possibility that the lower rate of survival of newly incorporated neurons in the GCL of mice injected with PS1 shRNA is the result of impaired morphology as a result of hastened maturation. We analyzed the morphology of mature neurons (GFP^+^BrdU+NeuN^+^) in the GCL using Sholl analysis of the arborization of neuronal dendritic trees. At 6 months after PS1 knockdown, we observed a significant decrease in the number of intersections with increasing distances from the soma (Fig 5a and 5b, F_(treatment)1,120_ = 12.51, ***P = 0.0002). This implies that the newly maturing neurons with reduced expression of PS1 have impaired connectivity compared to neurons with normal levels of PS1. To further investigate this idea, we next analyzed the number of dendritic spines (AutoSpine, MBF Biosciences) and found a significant decrease in the number of spines with increasing distance from the soma (Fig 5c, F_(treatment)1,120_ = 17.85, ***P<0.0001) and also in the mean number of spines per 10μm (*P<0.05). We found no significant differences in mean length, surface area, or volume (Fig 5e–5g) of dendritic branches. Taken together, these results indicate that PS1 downregulation induces premature neuronal differentiation that compromises neuronal morphology, leading to reduced dendritic branching and number of spines, which might be manifested by compromised function and reduced survival rate of these new neurons.

### PS1 Regulates Adult Hippocampal Neurogenesis via β-Catenin and Notch Signaling Pathways

**Fig 6:**
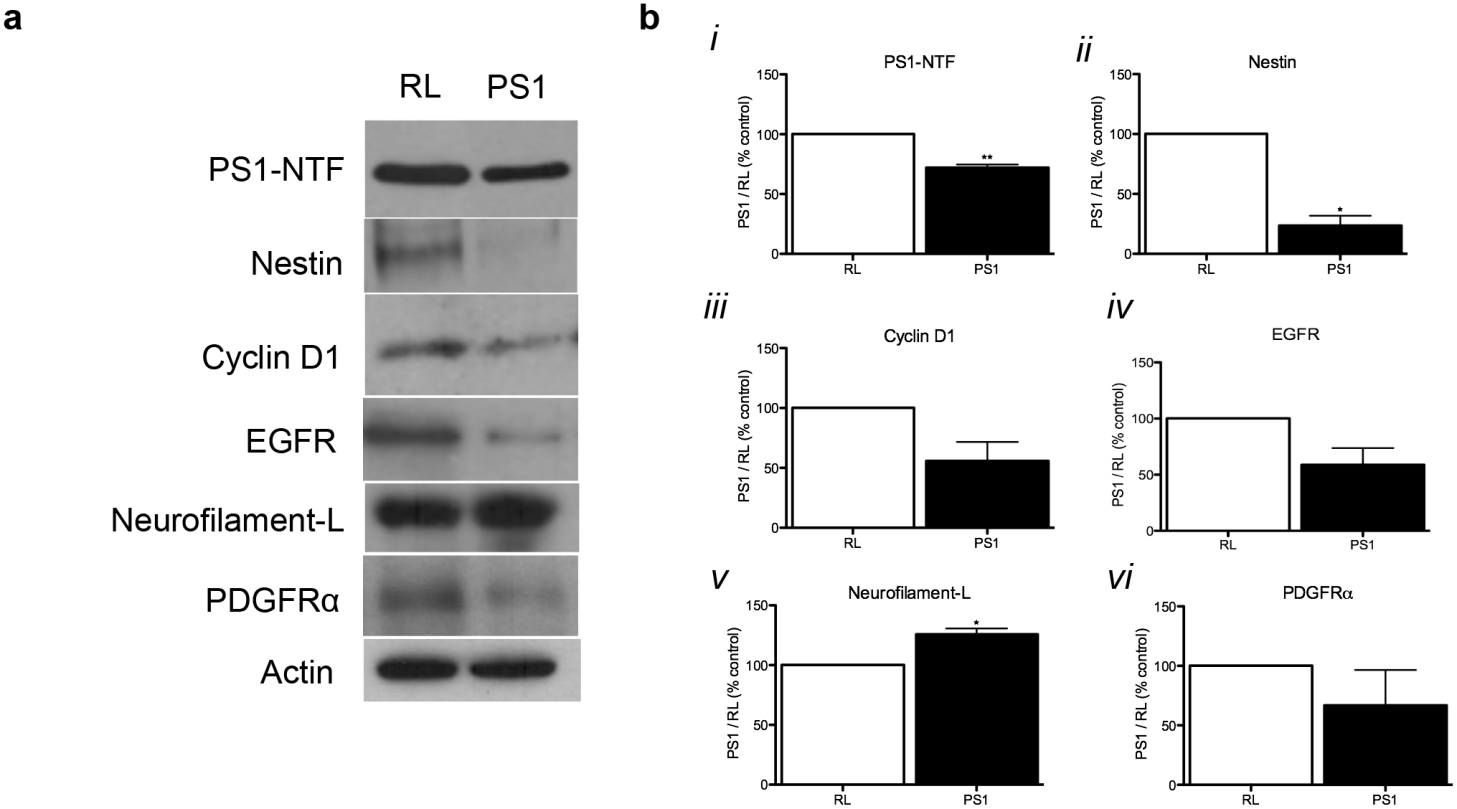
Downregulation of PS1 expression in adult neural progenitor cells induces the expression of differentiation markers. Quantitation of Western blot analysis reported as percentage of control values all normalized to actin. Western blot analysis (**A**) and quantification (**B**) of neurogenic signals in neural progenitor cell culture infected with lentiviral vectors expressing either control RL shRNA or PS1 shRNA. **(B*i-iii*)**. Expression of PS1-NTF (*i*), and Nestin (*ii*) are significantly reduced, while expression of Cyclin D1 (*iii*) and epidermal growth factor receptor (EGFR, *iv*) are slightly reduced following PS1 downregulation in protein extract of neurosphere cultures infected with lentivirus expressing PS1 shRNA. This suggests that reduced PS1 expression decreases proliferation and progenitor phase. Expression of Neurofilament-L (*v*) increased in neural progenitors infected with PS1 shRNA, supporting enhanced premature neuronal differentiation following PS1 downregulation in these cells. Levels of platelet derived growth factor receptor α (PDGFRα, *vi*) were not significantly different. Unpaired t-test with Welch’s correction, *P<0.05, **P<0.005. Error bars indicate ±SEM.

To examine the signaling pathways involved in PS1 regulation of neurogenesis, hippocampal neurospheres were infected with lentivirus expressing either PS1 shRNA or RL shRNA. Examination of PS1 protein expression 4 days after infection showed a significant reduction in expression of PS1-NTF by approximately 30% (Fig 6a and 6b*i*). Levels of nestin were significantly reduced, suggesting exit of NPCs from the progenitor stage following reduced PS1 expression (Fig 6a and 6b*ii*). In addition, levels of cyclin D1 (Fig 6a and 6b*iii*) and EGFR (Fig 6a and 6b*iv*) appear reduced, albeit not statistically significant. Levels of neurofilament-L, as an indicator of neuronal differentiation, are significantly increased (Fig 6a and 6b*v*). Interestingly, levels of PDGFRα were reduced, but did not reach significance (Fig 6a and 6b*vi*). These results support the phenotype we observed *in vivo*, in which following downregulation of PS1 in NPCs, they exit the cell cycle and differentiate into neurons and glia [13].

**Fig 7:**
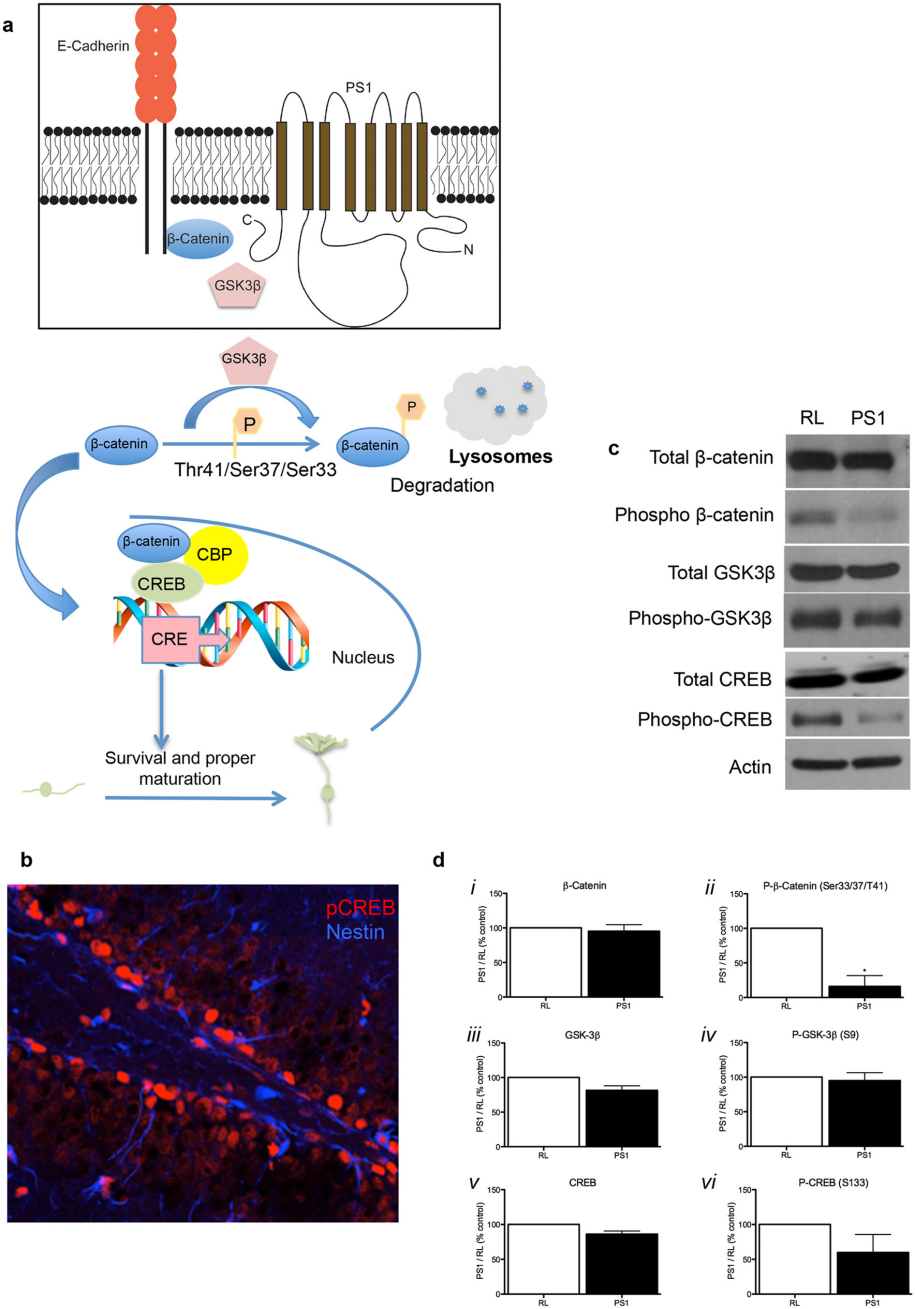
Downregulation of PS1 expression alters β-catenin metabolism and disrupts CREB expression. **A**. Schematics of neurogenic signals that interact with PS1. **B**. Confocal image (Zeiss LSM 510) representing colocalization of pCREB and Nestin in the dentate gyrus of the hippocampus (40x). **C, D**. (*i*) Expression of total β-Catenin were similar in both groups. However, levels of phospho-β-Catenin (*ii*) were significantly decreased in neural progenitor cells with reduced PS1 expression. Levels of total GSK3β (*iii*), phospho-GSK3β *(iv)* and CREB (*v*) were similar in both groups. Phospho-CREB (*vi*) shows decreasing trend, albeit statistically not significant. Western blot analysis significance was determined by unpaired t-test with Welch’s correction, *P<0.05, **P<0.01. Error bars indicate ±SEM.

Previously, we reported that reduction of PS1 expression in NPCs alters transcription level of β-catenin and notch-1 ligand delta-1 [13], but the functional significance of this reduction was unclear. Exploration preference (NOR) is mediated by β-catenin/Wnt signaling [15], and PS1 indirectly interacts with β-catenin and E-cadherin complex (Fig 7a) [16]. In addition, PS1 negatively regulates β-catenin stability by facilitating its phosphorylation by GSK3β on Thr41/Ser37/Ser33 leading to its degradation (Fig 7a) [17]. While levels of total β-catenin were not changed after PS1 knockdown (Fig 7c and 7d*i*), levels of phospho-β-catenin (Thr41/Ser37/Ser33) were significantly decreased (P<0.05, Fig 7*c* and 7*d* *ii*). This suggests that reduction in PS1 expression alters levels of phospho-β-catenin without affecting steady state levels of the protein.

Canonical Wnt signaling inactivates GSK3β leading to increased levels of cytoplasmic β-catenin, which then migrates to the nucleus and complexes with cAMP-response element-binding protein (CREB) binding protein (CBP) [18]. To address the role of GSK3β in PS1-dependent levels of phospho-β-catenin, we examined the expression of total GSK3β and its inactive form, phospho-GSK3β (Ser9). Neither levels of total GSK3β (Fig 7*c* and 7*d* *iii*) nor phospho-GSK3β (Ser9) were significantly changed following PS1 reduction. However, we observed a trend of reduced levels of GSK3β (Ser9) (Fig 7c and 7d*iv*, P = 0.0958), suggesting increased steady-state activity of GSK3β following PS1 knockdown. This may raise the possibility that alterations in levels of β-catenin phosphorylation are GSK3β- independent.

In addition to the canonical pathway, PS1 itself is a regulator of cAMP-response element-binding protein (CREB) activation. Specifically, the PS1-dependent cleavage product N-cadherin/carboxyl terminal fragment (N-Cad/CTF2) binds CBP and promotes its proteasomal degradation, inhibiting CREB activation [19]. Additionally, other studies suggest that PS1 indirectly regulates CREB expression and phosphorylation [20]. Phospho-CREB is critical for proper neuronal maturation [21]. Thus, we examined whether knockdown of PS1 would alter CREB expression or phosphorylation in NPCs. We observed that levels of total CREB were not changed (Fig 7c and 7d*v*). Interestingly, there was a trend towards reduced levels of CREB phosphorylation at Ser133 (pCREB-Ser133) following PS1 knockdown, albeit not statistically significant (P = 0.20; Fig 7c and 7d*vi*).

**Fig 8:**
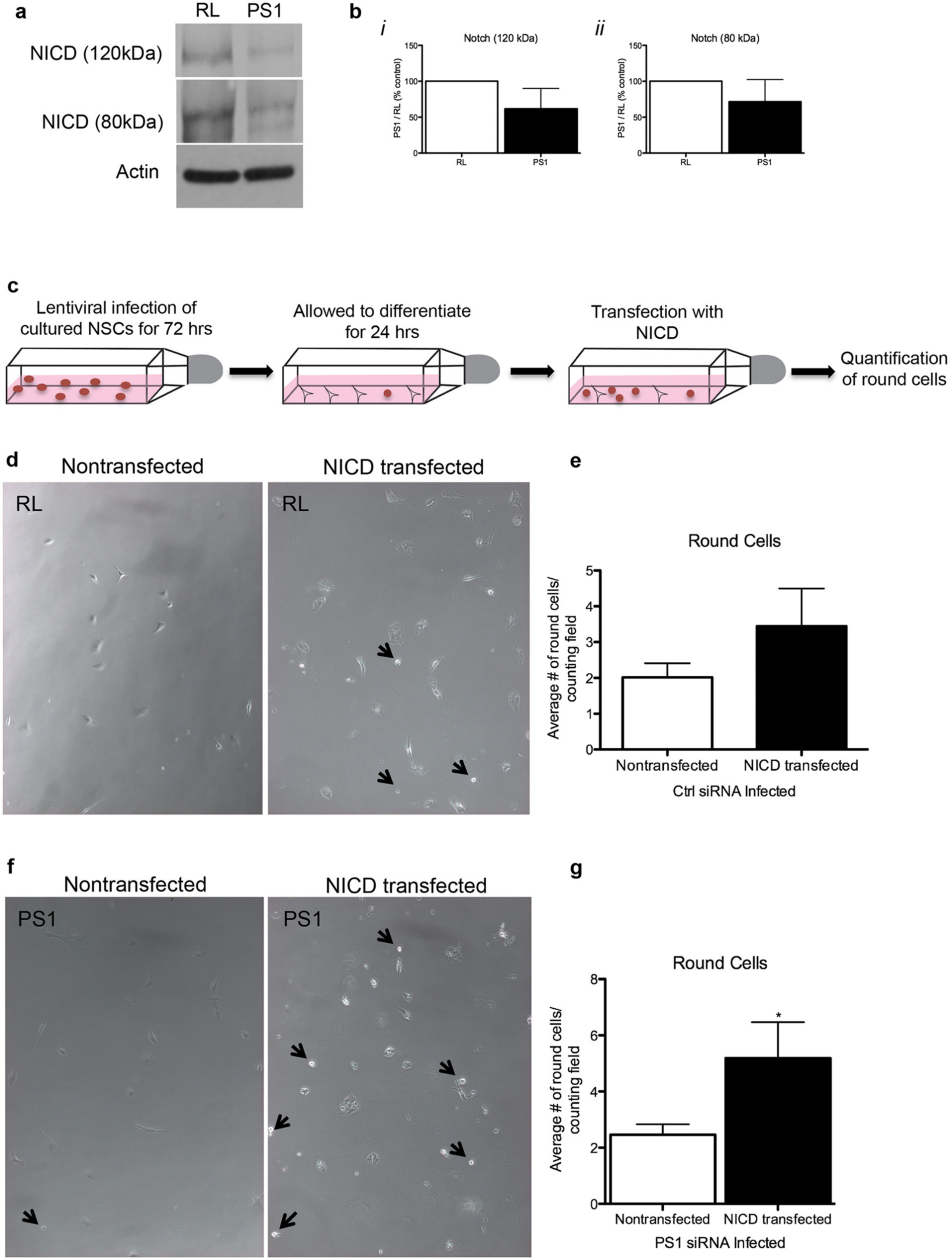
Compromised notch-1 cleavage and premature cell differentiation in neural progenitor cells expressing reduced levels of PS1 can be partially rescued by exogenous NICD. **A**. Reduced expression of PS1-NTF, NICD (80 & 120 kDa fragments) and PDGFRα in neural progenitor cells that were infected with lentiviral vectors expressing PS1 shRNA compared to cells infected with control RL shRNA. **B*i*-B*ii***. Densitometry analysis of Western blots. **C**. Scheme of infection of lentiviral vectors followed by NICD transfection. **D, F**. Phase contrast images of neural progenitor cells infected with lentiviral vectors expressing either control RL shRNA (D) or PS1 shRNA (F) followed by transfection with either control- or NICD-expressing vector. **E, G**. Quantification of round cells following NICD transfection show no change in the control condition (**E)** and a significant increase in the PS1 condition **(G)** indicating a reversal in morphological change following transfection with NICD-expressing vector with cells exhibiting round morphology (unpaired t-test; *P<0.05). Error bars indicate ±SEM.

Notch-1, a γ-secretase substrate is a central neurogenic signal that may play a role in PS1-regulated neurogenesis and differentiation of NPC. We observed a trend of reduced expression of both p120 and NICD fragments, albeit not statistically significant (Fig 8a, 8b*i* and 8b*ii* p = 0.0650, p = 0.1154 respectively). This may suggest reduced interaction of p120 with notch-1 ligand, leading to impaired intracellular signaling. It is difficult to assess the extent of reduced notch-1 metabolites necessary for NPC differentiation. Thus, to determine whether this reduction in notch-1 metabolites plays a role in the increased differentiation observed following reduced PS1 expression, we infected NPCs with lentivirus expressing either PS1 shRNA or RL shRNA lentivirus for 3 days, followed by transfection of the cells with NICD (Fig 8c). In agreement with previous observations, neurospheres infected with PS1 shRNA differentiated faster than neurospheres infected with RL shRNA [13]. Following transfection with NICD we observed a significant increase in the number of cells that acquired a round undifferentiated morphology compared to nontransfected cells in the PS1 shRNA infected NPCs (Fig 8d and 8e). The increase in the number of round undifferentiated cells compared to nontransfected cells in the RL shRNA infected NPCs were not statistically significant (Fig 8f and 8g). This may suggest that NICD plays a role in PS1-regulated NPC differentiation, and that the slight reduction in the expression of p120 and NICD may enhance their differentiation.

## Discussion

Here we show, for the first time, that downregulation of PS1 in adult NPCs and in new neurons in the dentate gyrus of the hippocampus induces cognitive deficits by promoting dysfunctional neuronal differentiation. Defective maturation is manifested by impaired dendritic arborization and reduced number of spines of new neurons. This is the first report to show that disrupting a protein linked to FAD exclusively in adult hippocampal neurogenesis can induce cognitive deficits. In addition, this is the first report to demonstrate that adult-born neurons have defective morphology following downregulation of PS1 expression.

Whether PS1 mutations cause loss or gain of γ-secretase function has been the subject of a long lasting debate [8, 19, 22]. There have been several recent studies supporting the hypothesis that *PSEN1* mutations by themselves can either impair (L435F) or cause complete loss of γ-secretase function (C410Y) [19, 23–25]. In the current study, we asked whether a reduction in PS1 expression in neural progenitor cells (NPCs) and new neurons in an otherwise intact dentate gyrus impairs hippocampal function in a neurogenesis-dependent manner.

Many of the FAD-mutant forms of *PSEN1* lead to some reduction in the proteolytic function of γ-secretase, while some mutants display only subtle reductions [26]. Our experimental design models the subtle loss of PS1 expression and function, which exhibits a very clear phenotype of premature and defective neuronal differentiation. Our *in vitro* data provides evidence that cells which are GFP^+^ PS1 shRNA display decreased expression of PS1 by approximately 30%. While technical limitations do not allow clear determination, it is reasonable to assume that the same extent of downregulation is observed *in vivo*. It should be noted that complete loss of function mutations in PS1 and other components of the γ-secretase complex (i.e., nicastrin and PEN-2) do not cause FAD or neurodegeneration [27]. Because APP is the only substrate of PS1 that can cause FAD, it is thought that amyloidosis underlies neurodegeneration in AD, and particularly the increased ratio of Aβ42/Aβ40. However, other studies suggest that loss of PS1 causes amyloidosis-free neurodegeneration [28, 29]. Our study supports the latter and suggests that reduction in PS1 level in NPCs and new neurons can cause cognitive deficits in the absence of amyloidosis.

Hippocampal NPCs mature into neurons that incorporate in the GCL of the DG throughout life. Impairment in their proper maturation and survival, as observed here, can progressively compromise hippocampal and specifically, DG function. In support, we show that reduced expression of PS1 in NPCs compromises pattern separation, the ability to discriminate between two similar contexts, a type of memory encoding in which adult neurogenesis plays a major role [14]. Mice injected with lentivirus expressing PS1 shRNA exhibit memory lapses that resemble first signs of mild cognitive impairment [30], suggesting that reduced PS1 expression in NPCs and new neurons in the dentate gyrus induces cognitive impairment. Mice that were tested 6 months after lentivirus injection performed worse overall than mice that were tested 3 months following injection, simply because they were older. Taking into account that age also plays a role in learning and memory, mice injected with PS1 shRNA could hardly learn the task, while mice injected with RL shRNA exhibited a similar pattern of behavior as the PS1 shRNA injected mice that were tested at 3 months after injection. This may suggest that reduced PS1 expression in NPCs and new neurons in the dentate gyrus of adult mice enhances age-linked memory deficits.

In addition to impairments in pattern separation, mice injected with lentivirus expressing PS1 shRNA into the SGL of the DG showed impaired performance in the hippocampus-dependent NOR test at 3 and 6 months post injection. By evaluating the ability of recognizing a previously presented stimulus, the NOR comprises the principal animal model test of human amnesia [31]. These results suggest that a moderate reduction of PS1 expression in NPCs and new neurons induces hippocampus-dependent learning and memory deficits in adult mice.

It should be taken into consideration that in spite of the time-dependent increase in the number of GFP^+^BrdU^+^NeuN^+^ in the GCL of injected wild type mice, it is very important to consider the possibility that some GFP^+^NeuN^+^ observed in the GCL of mice might be mature neurons that were infected by the lentivirus.

Our results show that downregulation of PS1 in GFP^+^NeuN^+^ neurons results in decreased dendritic branching and mean spine density. Defective morphology of mature granule neurons has potential detrimental effects on the circuits of the hippocampal formation. This may affect neurons in the entorhinal cortex that form synapses with these neurons, as well as compromise connections CA3 region pyramidal cells. Interestingly, previous studies document impaired dendritic branching and spines in dentate granule neurons in human brain sections of AD patients [32].

Our data suggests that of the neurogenic signals examined, downregulation of PS1 in NPCs induced the most dramatic reduction in phospho-β-catenin. Our previous study shows that PS1 downregulation results in decreased transcription of β-catenin mRNA [13]. Our current observation is that levels of β-catenin phosphorylation are dramatically reduced, while steady state levels of total β-catenin do not change. Taken together, this may suggest that stabilizing β-catenin compensates for reduced protein levels following PS1 downregulation. Our observation agrees with the effect of mutant PS1 causing a reduction in turnover of β-catenin [33]. Interference with β-catenin may impair Wnt signaling, a major regulator of adult hippocampal neurogenesis [34]. In addition, disrupting the interaction between β-catenin and E-cadherin through PS1 downregulation may result in deficits in the production of cell-cell adhesions that are critical for neural stem cell proliferation. This disruption would further manifest in an increase of differentiation, which has been shown by both our previous and current studies. Importantly, β-catenin/Wnt signaling is thought to mediate exploratory preference in the NOR task [15], supporting our observation that reduced PS1 in NPCs and new neurons compromised mouse performance in this task. Stabilizing β-catenin may interfere with its role in the initiation of gene transcription, and in agreement with previous observations [35], we find that compromised β-catenin phosphorylation is GSK3β activity-independent.

Notch-1 signaling is reported to play a role in regulation of adult neurogenesis in the hippocampus [36, 37] and subventricular zone [38]. It often keeps precursor cells in a dividing, unspecialized state [39]. Borghese *et al*. show that treatment of NSCs by the γ-secretase inhibitor DAPT (N-[N-(3,5-difluorophenacetyl)-L-alanyl]-S-phenylglycine t-butyl ester) results in an inhibition of Notch signaling (decreased NICD expression), leading to decreased proliferation and accelerated neuronal differentiation of these cells [40]. Our observation that the rescue of NICD levels reverses the differentiation phenotype is in full agreement with this study and provides further support for the mechanism by which PS1 regulates NPC differentiation. These data, combined with the current study, suggests that both the inhibition of γ-secretase directly, as well as a reduction in PS1 have similar effects [13, 40]. Thus, downregulation of this signaling pathway may underlie, at least in part, the premature differentiation of adult NPCs following PS1 downregulation. While we observed reduced levels of notch-1, albeit not statistically significant, it is not clear what NICD level mediates this effect. Taken together with the notion that levels of notch-1 in the adult brain are low, the role of notch-1 in PS1-regulated adult neurogenesis needs further investigation.

Likewise, while p-CREB plays a critical role in proper neuronal maturation [21], its role in PS1-regulated neurogenesis and neurogenesis-dependent learning and memory requires further investigation. Reduction in p-CREB levels may suggest compromised transcription of CRE-driven genes that support survival and differentiation of new neurons. Importantly, it has been reported that loss of CREB signaling can result in impaired dendritic arborization [21, 41].

In summary, this study shows molecular link between hippocampus-dependent learning and memory and PS1-regulated neurogenesis via β-catenin signaling pathway. This observation suggests that neurogenesis-based drugs may prevent or aid in the rescue cognitive deficits observed in Alzheimer’s disease.

## Materials and Methods

### Stereotaxic Injection of Lentivirus

All experiments were approved by the University of Illinois at Chicago Institutional Animal Care and Use Committee and performed accordingly. Lentivirus containing vectors expressing either shRNA for PS1 knockdown (4.11) or irrelevant control shRNA were produced as previously described [13]. Lentivirus was bilaterally injected into the SGL of 8-month-old male C57BL/6 mice (N = 11) using the following DG coordinates: AP -3.0 mm; ML ±2.0 mm; DV -2.5 mm. Mice were anesthetized using a mixture of ketamine (100 mg/kg) and xylazine (10 mg/kg). Their heads were shaved and the mice were fixed into the stereotaxic frame (Stoelting Co, Wood Dale, IL) in a skull flat orientation. The shaved area was cleaned 3 times with alternating 70% ethanol and betadine. A midline skin incision was made over the skull and tissue and periosteum was cleared to expose the skull surface. The skull was wiped with 30% hydrogen peroxide to dehydrate the bone to better highlight the location of Bregma. The injection site was determined relative to the Bregma according the mouse atlas of Paxinos and Franklin (2008) and a small burr hole was drilled. Lentivirus was then injected into the SGL at a rate of 0.2 μl/min for 5 minutes (total volume of 1 μl) using a 5 μl Hamilton syringe connected to an automatic injection pump. The syringe needle was then left in place for an additional minute to minimize virus diffusion up the needle track. The needle was slowly removed and the skin incision was closed. Mice were placed on a heating pad (PhysiTemp) until they recovered from anesthesia. The mice were individually housed until euthanasia either 3 months or 6 months following the surgery.

### Contextual Fear-Discrimination Learning

Neurogenesis-dependent learning and memory were evaluated using the pattern separation paradigm. This test has been performed as previously described [14]. Conditioning was conducted in two similar contexts: the shock-associated training context, A, and the similar (no-shock) context, B. Both test cages (17.8 x 17.8 x 30.5 cm), encased by isolation cubicle, had two clear Plexiglas walls, two grey metal walls and a stainless steel grid floor (Coulbourn Instruments). In cage A (training context) the house light and fan were turned on. A mild lemon blossom scent was used as an olfactory cue, and 70% ethanol was used to clean grids between runs. Test cage B (similar context) differed from test cage A in that the metal walls had black and white inserts, the house light and fan were turned off and the chamber door was left ajar. A mild peppermint scent was used as an olfactory cue, and Clorox disinfecting wipes were used to clean the grids between runs. Mouse motion was recorded by a digital video camera mounted above the test cage (Coulbourn Instruments). On day 0 mice were exposed only to the training context. For the next nine consecutive days (days 1–9) mice were exposed to the training context and the similar context in a randomized order. In test cage A they received a single 2 second foot shock of 0.75 mA at 185 seconds after placement in the cage and remained in the cage for an additional 15 seconds. After 1 hour, mice were placed in test cage B, in which they were left for 180 seconds and were never shocked. FreezeFrame and FreezeView software (Actimetrics) were used for recording and analyzing freezing behavior, respectively. Percentage of freezing during the first 180 seconds in each context for each day was computed. Discrimination ratio was calculated as (% Freezing A − % Freezing B) / (% Freezing A + % Freezing B). Statistical analysis was done using one-way (context) repeated measures ANOVA over days for each treatment and followed by paired t-test between contexts in each day. Paired t-test was chosen due to the smaller variance within subjects than within group.

### Novel Object Recognition Test

This test was performed as previously described [31, 42]. On the first day mice were placed in the arena (Plexiglas 45 x 30 x 15 cm) for 1 hour of habituation. On the following day, in the familiarization phase, mice were placed in the arena with two identical objects and allowed to explore them for 5 minutes. Four hours later, in the test phase, they were reintroduced into the arena in which one of the identical objects was replaced with a novel object. The mice were then allowed to explore the objects for 5 minutes. Identical and novel objects were similar in size but differed in color and shape. The location of the objects (identical-identical or identical-novel) was randomized to avoid side preference. Object exploration time was recorded manually and the percentage of exploration time of each of the objects was computed. Exploration time of the novel object vs. the familiar object during the test phase was compared and analyzed by unpaired t-test.

### BrdU Injections

A single dose of 5’-bromo-2’-deoxyuridine (BrdU; Sigma) was administered intraperitoneally (100 mg/kg), in physiological saline. Three weeks following BrdU injection animals were anesthetized via isoflurane overdose and transcardially perfused with ice cold 1X PBS followed by 4% paraformaldehyde (PFA). The brains were then removed, dissected into two hemispheres and post-fixed in 4% PFA for 48 hours at 4°C. They were then were cryoprotected in 30% sucrose and stored at 4°C.

### Antibodies

For quantification of specific GFP positive cell populations, the following triple labeling was performed: mature neurons—mouse anti-GFP (1:200; Santa Cruz Biotechnology), chicken anti-β-III tubulin (1:100; Millipore), and rat anti-BrdU (1:400; Accurate Chemical & Scientific Corp.); neuroblasts—mouse anti-GFP, goat anti-doublecortin (DCX; 1:400; Santa Cruz Biotechnology), and rat anti-BrdU; progenitor cells—mouse anti-GFP, rat anti-BrdU, rabbit anti-nestin (1:200; Millipore); stem cells- goat anti-GFP, mouse anti-nestin, rabbit anti-GFAP (1:500; Dako); mature astrocytes—goat anti-GFP, mouse anti-GFAP, rabbit anti-S100ß (1:3000, Abcam). The following secondary antibodies were used from Jackson ImmunoResearch Laboratories (West Grove, PA): biotinylated species-specific anti-IgG (all used at 1:250), Cy3-conjugated Donkey anti-Rat (1:500), Alexa Fluor 647 (AF647)-conjugated Donkey anti-Mouse (1:250), Cy2-conjugated Streptavidin (1:250), DAPI Nucleic Acid Stain (1:10,000, Life Technologies, Grand Island, NY).

### Immunohistochemistry

50 μm sagittal sections were cut using a sliding freezing microtome (Leica Biosystems, Buffalo Grove, IL) and were stored in cryoprotectant (glycerol, ethylene glycol, 1X PBS) at -20°C. Every sixth section was used for immunohistochemistry. Floating sections were first rinsed 3 times in 1X TBS. For BrdU antigen retrieval, sections were subjected to pretreatment with 2N HCl solution for 30 min at 37°C. Next, sections were incubated in 0.1 M borate buffer for 10 min at room temperature to neutralize any remaining acid and were rinsed 6 times in 1X TBS for 10 minutes each wash. Sections were blocked for 1 hour at room temperature in 1X TBS, 5% normal donkey serum, 0.25% Triton X-100 (TBS^++^). Sections were then incubated in primary antibodies diluted in 1X TBS, 0.25% Triton X-100 (TBS^+^) for 72 h on an orbital shaker at 4°C. Following two additional 1 hour blocking steps at room temperature, sections were incubated with biotinylated species-specific anti-IgG in TBS^+^ for 1 hour at room temperature. Sections were then incubated in fluorophore conjugated secondary antibodies diluted in TBS^+^ for 2 hours at room temperature. A final incubation in 1X TBS containing DAPI for 5 minutes at room temperature was performed before sections were mounted onto SuperFrost Plus glass slides and cover slipped using polyvinyl acetate-1,4-diazabicyclo-[2.2.2]octane (PVA-DABCO, Sigma Aldrich, St. Louis, MO). Slides were dried overnight at room temperature in the dark and then stored at 4°C.

### Stereology

Quantification of immunostained sections was done using Stereo Investigator (MicroBrightField Bioscience). Immunostained sections were counted using the following parameters: the counting frames as well as the sampling grid were set to 100 μm x 100 μm. The section thickness was averaged at 35 μm. The subgranular layer and the granular layer of the dentate gyrus were counted.

### Neurosphere Culture

Subventricular zone derived neural stem cells were isolated as previously described [43]. Adult male C57BL/6 mice (2–4 months of age, 3–4 per group) were sacrificed via isoflurane overdose followed by cervical dislocation. Their brains were immediately removed and dissected into two hemispheres. Hemispheres were then cut at approximately -0.94 mm relative to Bregma, according to an adult mouse brain atlas [44]. Tissue samples were minced with a scalpel and incubated in 0.1% trypsin—EDTA (Invitrogen) for 7 min at 37°C. The enzymatic digestion was quenched with 3 parts 0.014% w/v trypsin inhibitor (Sigma) in HBSS (Life Technologies). After centrifugation, the pellet was resuspended in 1 mL of medium and mechanically triturated to disassociate the cells. The cells were then centrifuged at 700 rpm and resuspended in 500 μl of medium. Viable cells were counted on a hemocytometer using trypan blue exclusion (Sigma). Hippocampal cells were seeded at a density of 10,000 cells/cm^2^ in non-tissue culture-treated 24-well plates (BD Biosciences) with 2 mL of medium per well containing 20 ng/mL human recombinant epidermal growth factor (EGF) and 10 ng/mL mouse recombinant FGF-2 (BD Biosciences). Medium consisted of DMEM/F-12 medium, supplemented with N2, B27, penicillin/streptomycin (Life Technologies), and 2 ρg/mL heparin (Sigma). Cells were incubated for 10 days in 37°C + 5% CO2 to permit primary neurosphere formation. Cells were fed with growth factors every 3 days.

### Infection of Neurosphere Cultures with Lentivirus

Adult neurospheres cultured from the subventricular zone were used for infections between passages 2 to 4. Neurospheres were collected, centrifuged, and enzymatically digested with 0.05% Trypsin-EDTA for 5 min at 37°C. Trypsin inhibitor was added and cells were again centrifuged at 1000rpm for 5 minutes. The pellet was suspended in complete growth medium and cells were then counted and seeded in a concentration of 10,000 cell per cm^2^. Infection media consisted of non-concentrated virus expressing either PS1 shRNA 1.1 or irrelevant shRNA in complete neurosphere growth media. Infection was carried out for 3 days and GFP expression was confirmed using inverted microscope (Olympus) and fluorescence illumination (Lumen Dynamics).

### Transfection of PS1 shRNA-Infected NPCs with NICD

Neurospheres were prepared and infected with either PS1 shRNA 1.1 or irrelevant shRNA as described above. The cells were then collected and plated 10,000 cells per well in a 24 well plate in differentiation media (growth media plus 5% FBS and no growth factors added). Following 24 hours of differentiation, the cells were transfected using lipofectamine LTX reagent (Life Technologies, Grand Island, NY) with either 1 μg of NICD DNA (kindly provided by Dr. Kamal Sharma, UIC) or were not transfected with any DNA. The cells were transfected for 24 hours then analyzed in the culture dish using an inverted microscope (Olympus) and fluorescent illumination (Lumen Dynamics). Each well was divided into four quadrants and GFP positive cells were counted in two randomly selected locations from each quadrant. The total number of cells, the number of cells with processes, and the number of round, progenitor-like cells were quantified.

### Immunocytochemistry

Following infection with either PS1 or RL shRNA, cells were harvested and plated on matrigel and allowed to differentiate for 5 days Differentiated cells were fixed using 4% PFA for 20 min at room temperature. Following three washes in TBS cells were blocked in TBS^++^ for 30 min. Cells were then incubated in primary antibodies diluted in TBS^+^ for 2 h at room temperature and blocked again in TBS^++^ for 30 min. Cells were then incubated in biotinylated species-specific anti-IgG in TBS^+^ for 30 min. Cells were then incubated in fluorophore conjugated secondary antibodies diluted in TBS^+^ for 1 h at room temperature. Cells were then incubated in a DAPI solution for 5 minutes, washed, and mounted using PVA-DABCO and left to dry overnight at room temperature. Slides were stored at 4°C.

### Neuronal Morphology Analysis

For neuronal morphology analysis, a total of 16 cells (GFP^+^NeuN^+^) per group (RL shRNA vs. PS1 shRNA) were imaged on a Zeiss LSM 510 confocal microscope. Z-stacks were compressed, processed in Neurolucida, and Sholl analysis was completed using AutoNeuron (MBF Biosciences). Concentric circles were drawn by the AutoNeuron software every 10μm away from the soma and the number of intersections (dendritic branches) with these circles was quantified. Spine density was analyzed in the same manner, using the AutoSpine extension in the Neurolucida software (MBF Biosciences).

### Western Blot Analysis

**I**nfected neurospheres cultured from the subventricular zone were collected and washed twice with cold TBS. Protein was extracted using immunoprecipitation buffer (150 mM NaCl, 50 mM Tris-HCl, 0.5% Triton X-100, 0.5% sodium deoxycholate, 5mM EDTA, 1% SDS, mammalian Protease Inhibitor Cocktail II [Sigma], 100mM phenylmethylsulfonyl fluoride [PMSF; Sigma], and phosphatase inhibitors; 2 mM Na-Orthovanadate, 50 nM RR-Microcystin, 50 nM Okadaic acid, 100 nM K252a, 100 nM Staurosporine [Sigma] and 100 mM Potassium phosphate cocktail II [Calbiochem]). The pellet was incubated in the buffer for 10 min on ice and then sonicated for 30 sec. Nuclei and cell debris were removed by 5 min centrifugation at 1000rpm at 4°C. Protein concentration was quantified using the BCA (bicinchoninic acid) method (Pierce). Equal amounts (50 μg) of protein were taken for immunoblotting.

### Statistics

All statistical analysis was used using Prism 5 (Graphpad Software, Inc., La Jolla, CA). *Fear conditioning* behavioral data was analyzed using two-way ANOVA with repeated measures (context) over days for each treatment. Post-hoc comparisons were used between contexts and treatments on days that were significant in the recognition memory paradigm (paired *t* test, *p<0.05). Paired *t* test was chosen due to the smaller variance within subjects than within group. *Novel object recognition* behavioral data was analyzed using paired two-tailed *t* test (*p<0.05). *Stereology data* was analyzed using unpaired one-tailed *t* test with Welch’s Correction due to unequal variance (*p<0.05). Dendritic branching and spine density analysis was done using Autoneuron and Autospine (MBF Biosciences), respectively, which measures three-dimensional image volume stacks. *Sholl Analysis* was completed using two-way ANOVA with repeated measures (distance from soma) comparing number of intersections and distance away from the soma. Post-hoc comparisons were used between number of intersections and distance away from the soma at distances that were significant (*p<0.05). *Mean length*, *volume*, *and surface area measurements* were analyzed using unpaired one-tailed *t* test with Welch’s correction for unequal variance. *Spine density* analysis was completed using two-way ANOVA with repeated measures (distance from soma) comparing number of spines with increasing distance from the soma. Post-hoc comparisons were used between number of spines and distance away from the soma at distances that were significant (*p<0.05). *Mean spine density* was compared using unpaired one-tailed *t* test with Welch’s correction for unequal variance (*p<0.05). *NICD transfection round cell* analysis was completed using an unpaired, two-tailed t test (*p<0.05).

## Supporting Information

S1 Fig No change in the number of neural progenitor cells in the subgranular layer 3 months following injection. **A*i-iii***. No change in the number of new immature neurons (A*i*), new non-neuronal proliferating (A*ii*), or immature neurons (A*iii*). **B*i-iii***. No significant change in the type I NSCs, type II NPCs (B*ii*), or mature astrocytes (B*iii*). Unpaired t-test with Welch’s Correction, *P<0.05. Error bars indicate ±SEM.
(TIF)

## References

[1] Aimone JB, Li Y, Lee SW, Clemenson GD, Deng W, Gage FH. Regulation and function of adult neurogenesis: from genes to cognition. Physiological reviews. 2014;94(4):991–1026. 10.1152/physrev.00004.2014 25287858 PMC4280160

[2] Spalding KL, Bergmann O, Alkass K, Bernard S, Salehpour M, Huttner HB, et al Dynamics of hippocampal neurogenesis in adult humans. Cell. 2013;153(6):1219–27. 10.1016/j.cell.2013.05.002 23746839 PMC4394608

[3] Aimone JB, Deng W, Gage FH. Resolving new memories: a critical look at the dentate gyrus, adult neurogenesis, and pattern separation. Neuron. 2011;70(4):589–96. Epub 2011/05/26. 10.1016/j.neuron.2011.05.010 21609818 PMC3240575

[4] Aimone JB, Deng W, Gage FH. Adult neurogenesis: integrating theories and separating functions. Trends Cogn Sci. 2010;14(7):325–37. Epub 2010/05/18. 10.1016/j.tics.2010.04.003 20471301 PMC2904863

[5] Hyman BT, Phelps CH, Beach TG, Bigio EH, Cairns NJ, Carrillo MC, et al National Institute on Aging-Alzheimer's Association guidelines for the neuropathologic assessment of Alzheimer's disease. Alzheimers Dement. 2012;8(1):1–13. Epub 2012/01/24. 10.1016/j.jalz.2011.10.007 22265587 PMC3266529

[6] De Strooper B, Saftig P, Craessaerts K, Vanderstichele H, Guhde G, Annaert W, et al Deficiency of presenilin-1 inhibits the normal cleavage of amyloid precursor protein. Nature. 1998;391(6665):387–90. 9450754 10.1038/34910

[7] De Strooper B, Annaert W, Cupers P, Saftig P, Craessaerts K, Mumm JS, et al A presenilin-1-dependent gamma-secretase-like protease mediates release of Notch intracellular domain. Nature. 1999;398(6727):518–22. 10206645 10.1038/19083

[8] De Strooper B, Iwatsubo T, Wolfe MS. Presenilins and gamma-secretase: structure, function, and role in Alzheimer Disease. Cold Spring Harb Perspect Med. 2012;2(1):a006304 Epub 2012/02/09. 10.1101/cshperspect.a006304 22315713 PMC3253024

[9] Herreman A, Serneels L, Annaert W, Collen D, Schoonjans L, De Strooper B. Total inactivation of gamma-secretase activity in presenilin-deficient embryonic stem cells. Nature cell biology. 2000;2(7):461–2. 10878813 10.1038/35017105

[10] Wolfe MS, Xia W, Ostaszewski BL, Diehl TS, Kimberly WT, Selkoe DJ. Two transmembrane aspartates in presenilin-1 required for presenilin endoproteolysis and gamma-secretase activity. Nature. 1999;398(6727):513–7. 10206644 10.1038/19077

[11] Li YM, Lai MT, Xu M, Huang Q, DiMuzio-Mower J, Sardana MK, et al Presenilin 1 is linked with gamma-secretase activity in the detergent solubilized state. Proceedings of the National Academy of Sciences of the United States of America. 2000;97(11):6138–43. 10801983 10.1073/pnas.110126897 PMC18571

[12] Zhang Z, Nadeau P, Song W, Donoviel D, Yuan M, Bernstein A, et al Presenilins are required for gamma-secretase cleavage of beta-APP and transmembrane cleavage of Notch-1. Nature cell biology. 2000;2(7):463–5. 10878814 10.1038/35017108

[13] Gadadhar A, Marr RA, Lazarov O. Presenilin-1 regulates neural progenitor cell differentiation in the adult brain. J Neurosci. 2011;31(7):2615–23. Epub Feb 16. 10.1523/JNEUROSCI.4767-10.2011 21325529 PMC3050597

[14] Sahay A, Scobie KN, Hill AS, O'Carroll CM, Kheirbek MA, Burghardt NS, et al Increasing adult hippocampal neurogenesis is sufficient to improve pattern separation. Nature. 2011;472(7344):466–70. Epub 2011/04/05. 10.1038/nature09817 21460835 PMC3084370

[15] Fortress AM, Schram SL, Tuscher JJ, Frick KM. Canonical Wnt signaling is necessary for object recognition memory consolidation. J Neurosci. 2013;33(31):12619–26. Epub 2013/08/02. 10.1523/JNEUROSCI.0659-13.2013 23904598 PMC6618550

[16] Marambaud P, Shioi J, Serban G, Georgakopoulos A, Sarner S, Nagy V, et al A presenilin-1/gamma-secretase cleavage releases the E-cadherin intracellular domain and regulates disassembly of adherens junctions. Embo J. 2002;21(8):1948–56. 11953314 10.1093/emboj/21.8.1948 PMC125968

[17] Serban G, Kouchi Z, Baki L, Georgakopoulos A, Litterst CM, Shioi J, et al Cadherins mediate both the association between PS1 and beta-catenin and the effects of PS1 on beta-catenin stability. J Biol Chem. 2005;280(43):36007–12. 16126725 10.1074/jbc.M507503200 PMC4005066

[18] Ciani L, Salinas PC. WNTs in the vertebrate nervous system: from patterning to neuronal connectivity. Nat Rev Neurosci. 2005;6(5):351–62. Epub 2005/04/16. 15832199 10.1038/nrn1665

[19] Marambaud P, Wen PH, Dutt A, Shioi J, Takashima A, Siman R, et al A CBP binding transcriptional repressor produced by the PS1/epsilon-cleavage of N-cadherin is inhibited by PS1 FAD mutations. Cell. 2003;114(5):635–45. Epub 2003/09/19. 13678586 10.1016/j.cell.2003.08.008

[20] Watanabe H, Smith MJ, Heilig E, Beglopoulos V, Kelleher RJ 3rd, Shen J. Indirect regulation of presenilins in CREB-mediated transcription. J Biol Chem. 2009;284(20):13705–13. Epub 2009/03/18. 10.1074/jbc.M809168200 19289467 PMC2679472

[21] Jagasia R, Steib K, Englberger E, Herold S, Faus-Kessler T, Saxe M, et al GABA-cAMP response element-binding protein signaling regulates maturation and survival of newly generated neurons in the adult hippocampus. J Neurosci. 2009;29(25):7966–77. Epub 2009/06/26. 10.1523/JNEUROSCI.1054-09.2009 19553437 PMC2776747

[22] Chavez-Gutierrez L, Bammens L, Benilova I, Vandersteen A, Benurwar M, Borgers M, et al The mechanism of gamma-Secretase dysfunction in familial Alzheimer disease. EMBO J. 2012;31(10):2261–74. Epub 2012/04/17. 10.1038/emboj.2012.79 22505025 PMC3364747

[23] Xia D, Watanabe H, Wu B, Lee SH, Li Y, Tsvetkov E, et al Presenilin-1 Knockin Mice Reveal Loss-of-Function Mechanism for Familial Alzheimer's Disease. Neuron. 2015;85(5):967–81. 10.1016/j.neuron.2015.02.010 25741723 PMC4358812

[24] Heilig EA, Xia W, Shen J, Kelleher RJ 3rd. A presenilin-1 mutation identified in familial Alzheimer disease with cotton wool plaques causes a nearly complete loss of gamma-secretase activity. The Journal of biological chemistry. 2010;285(29):22350–9. 10.1074/jbc.M110.116962 20460383 PMC2903357

[25] Heilig EA, Gutti U, Tai T, Shen J, Kelleher RJ 3rd. Trans-dominant negative effects of pathogenic PSEN1 mutations on gamma-secretase activity and Abeta production. The Journal of neuroscience: the official journal of the Society for Neuroscience. 2013;33(28):11606–17. 10.1523/JNEUROSCI.0954-13.2013 23843529 PMC3724549

[26] Kulic L, Walter J, Multhaup G, Teplow DB, Baumeister R, Romig H, et al Separation of presenilin function in amyloid beta-peptide generation and endoproteolysis of Notch. Proc Natl Acad Sci U S A. 2000;97(11):5913–8. Epub 2000/05/17. 10811883 10.1073/pnas.100049897 PMC18533

[27] Wang B, Yang W, Wen W, Sun J, Su B, Liu B, et al Gamma-secretase gene mutations in familial acne inversa. Science. 2010;330(6007):1065 Epub 2010/10/12. 10.1126/science.1196284 20929727

[28] Saura CA, Choi SY, Beglopoulos V, Malkani S, Zhang D, Shankaranarayana Rao BS, et al Loss of presenilin function causes impairments of memory and synaptic plasticity followed by age-dependent neurodegeneration. Neuron. 2004;42(1):23–36. Epub 2004/04/07. 15066262 10.1016/s0896-6273(04)00182-5

[29] Chen Q, Nakajima A, Choi SH, Xiong X, Tang YP. Loss of presenilin function causes Alzheimer's disease-like neurodegeneration in the mouse. J Neurosci Res. 2008;86(7):1615–25. Epub 2008/01/15. 10.1002/jnr.21601 18189321

[30] Albert MS, DeKosky ST, Dickson D, Dubois B, Feldman HH, Fox NC, et al The diagnosis of mild cognitive impairment due to Alzheimer's disease: recommendations from the National Institute on Aging-Alzheimer's Association workgroups on diagnostic guidelines for Alzheimer's disease. Alzheimers Dement. 2011;7(3):270–9. Epub 2011/04/26. 10.1016/j.jalz.2011.03.008 21514249 PMC3312027

[31] Antunes M, Biala G. The novel object recognition memory: neurobiology, test procedure, and its modifications. Cognitive processing. 2012;13(2):93–110. Epub 2011/12/14. 10.1007/s10339-011-0430-z 22160349 PMC3332351

[32] Einstein G, Buranosky R, Crain BJ. Dendritic pathology of granule cells in Alzheimer's disease is unrelated to neuritic plaques. J Neurosci. 1994;14(8):5077–88. Epub 1994/08/01. 8046469 10.1523/JNEUROSCI.14-08-05077.1994 PMC6577187

[33] Kang DE, Soriano S, Frosch MP, Collins T, Naruse S, Sisodia SS, et al Presenilin 1 facilitates the constitutive turnover of beta-catenin: differential activity of Alzheimer's disease-linked PS1 mutants in the beta-catenin-signaling pathway. J Neurosci. 1999;19(11):4229–37. Epub 1999/05/26. 10341227 10.1523/JNEUROSCI.19-11-04229.1999 PMC6782616

[34] Lie DC, Colamarino SA, Song HJ, Desire L, Mira H, Consiglio A, et al Wnt signalling regulates adult hippocampal neurogenesis. Nature. 2005;437(7063):1370–5. 16251967 10.1038/nature04108

[35] Palacino JJ, Murphy MP, Murayama O, Iwasaki K, Fujiwara M, Takashima A, et al Presenilin 1 regulates beta-catenin-mediated transcription in a glycogen synthase kinase-3-independent fashion. J Biol Chem. 2001;276(42):38563–9. Epub 2001/08/16. 11504726 10.1074/jbc.M105376200

[36] Ables JL, Decarolis NA, Johnson MA, Rivera PD, Gao Z, Cooper DC, et al Notch1 is required for maintenance of the reservoir of adult hippocampal stem cells. J Neurosci. 2010;30(31):10484–92. Epub 2010/08/06. 10.1523/JNEUROSCI.4721-09.2010 20685991 PMC2935844

[37] Lavado A, Oliver G. Jagged1 is necessary for postnatal and adult neurogenesis in the dentate gyrus. Dev Biol. 2014;388(1):11–21. Epub 2014/02/18. 10.1016/j.ydbio.2014.02.004 24530424 PMC4009513

[38] Sun F, Mao X, Xie L, Ding M, Shao B, Jin K. Notch1 signaling modulates neuronal progenitor activity in the subventricular zone in response to aging and focal ischemia. Aging Cell. 2013;12(6):978–87. Epub 2013/07/10. 10.1111/acel.12134 23834718 PMC3838489

[39] Shin J, Poling J, Park HC, Appel B. Notch signaling regulates neural precursor allocation and binary neuronal fate decisions in zebrafish. Development. 2007;134(10):1911–20. Epub 2007/04/20. 17442701 10.1242/dev.001602

[40] Borghese L, Dolezalova D, Opitz T, Haupt S, Leinhaas A, Steinfarz B, et al Inhibition of notch signaling in human embryonic stem cell-derived neural stem cells delays G1/S phase transition and accelerates neuronal differentiation in vitro and in vivo. Stem Cells. 2010;28(5):955–64. Epub 2010/03/18. 10.1002/stem.408 20235098

[41] Dayer AG, Ford AA, Cleaver KM, Yassaee M, Cameron HA. Short-term and long-term survival of new neurons in the rat dentate gyrus. J Comp Neurol. 2003;460(4):563–72. Epub 2003/04/30. 12717714 10.1002/cne.10675

[42] Balderas I, Rodriguez-Ortiz CJ, Salgado-Tonda P, Chavez-Hurtado J, McGaugh JL, Bermudez-Rattoni F. The consolidation of object and context recognition memory involve different regions of the temporal lobe. Learn Mem. 2008;15(9):618–24. Epub 2008/08/30. 10.1101/lm.1028008 18723431 PMC2632790

[43] Bonaguidi MA, Peng CY, McGuire T, Falciglia G, Gobeske KT, Czeisler C, et al Noggin expands neural stem cells in the adult hippocampus. J Neurosci. 2008;28(37):9194–204. Epub 2008/09/12. 10.1523/JNEUROSCI.3314-07.2008 18784300 PMC3651371

[44] Franklin KBJ and Paxinos G, The mouse brain in stereotaxic coordinates, 3rd Edition Academic Press, Elsevier 2008 ISBN 9780123742445.

