# Supplementary figures and images for: Presenilin-1 Dependent Neurogenesis Regulates Hippocampal Learning and Memory
Source: PLoS One. 2015 Jun 22;10(6):e0131266. doi: 10.1371/journal.pone.0131266 (PMC4476567; doi:10.1371/journal.pone.0131266)

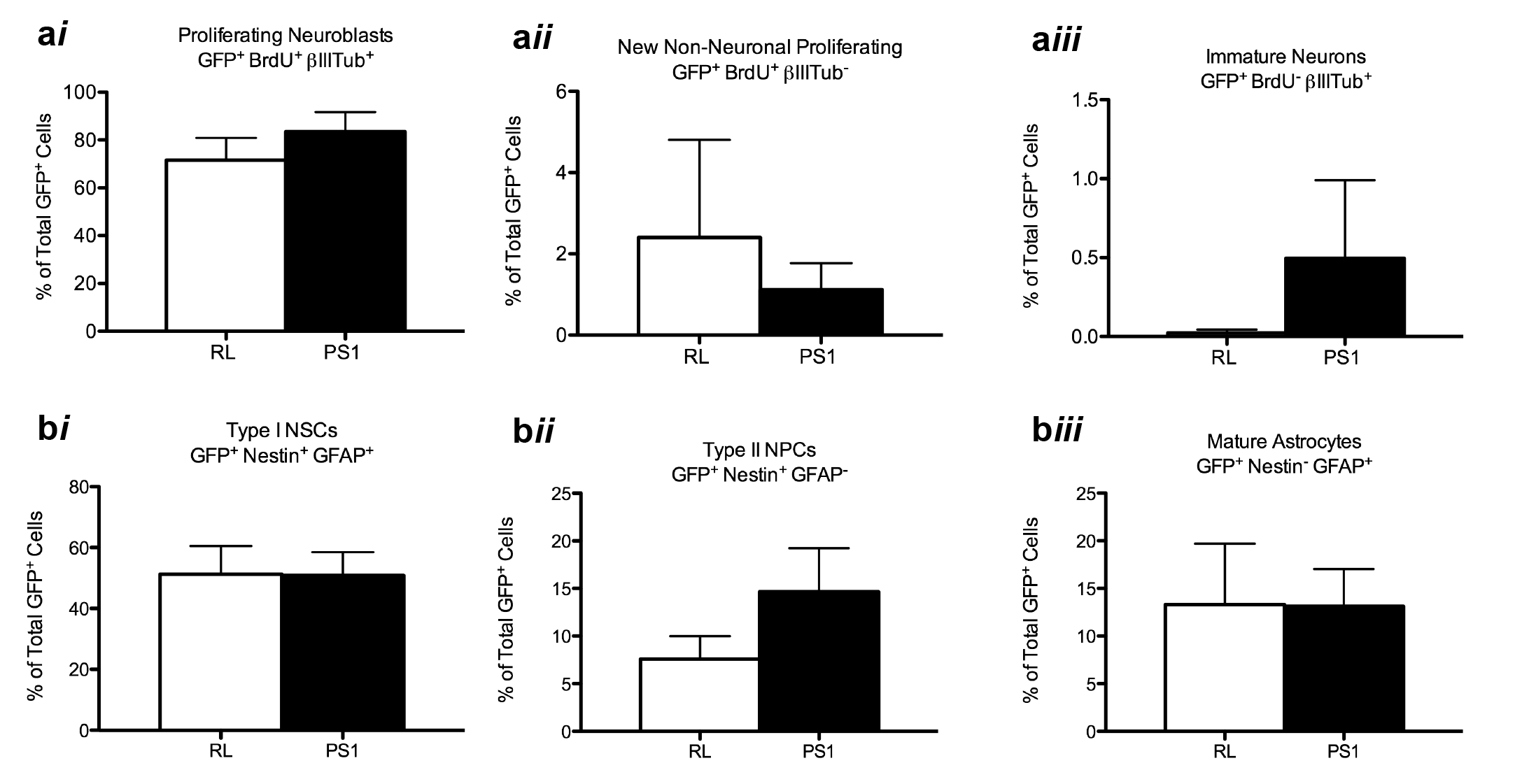

Supplement: S1 Fig — Ai-iii. No change in the number of new immature neurons (Ai), new non-neuronal proliferating (Aii), or immature neurons (Aiii). Bi-iii. No significant change in the type I NSCs, type II NPCs (Bii), or mature astrocytes (Biii). Unpaired t-test with Welch’s Correction, *P<0.05. Error bars indicate ±SEM. (TIF) [file pone.0131266.s001.tif]
